# Associations between gut microbiota on carcass traits and meat quality in Neijiang pigs, Yorkshire pigs, and their hybrids

**DOI:** 10.7717/peerj.21663

**Published:** 2026-09-04

**Authors:** Ziling Hao, Yiting Yang, Zhijuan Yan, Yong Du, Junxiang Mao, Lei Chen, Ye Zhao, Lili Niu, Xiaofeng Zhou, Linyuan Shen, Mailin Gan, Li Zhu

**Affiliations:** 1 State Key Laboratory of Swine and Poultry Breeding Industry, Sichuan Agricultural University, Cheng’du, China; 2 Key Laboratory of Livestock and Poultry Multi-Omics, Ministry of Agriculture and Rural Affairs, College of Animal and Technology, Sichuan Agricultural University, Cheng’du, China; 3 Farm Animal Germplasm Resources and Biotech Breeding Key Laboratory of Sichuan Province, Sichuan Agricultural University, Cheng’du, China

**Keywords:** Pig, Carcass characteristic, Meat quality, 16S rRNA, Cross-breeding

## Abstract

This study was designed as an exploratory analysis to compare carcass performance, meat quality traits, and gut microbiota of Neijiang pigs (NN), Yorkshire pigs (YY), and Yorkshire × Neijiang hybrid pigs (YN), with the goal of generating testable hypotheses regarding potential links between gut microbial composition and production phenotypes. Compared with NN pigs, YN hybrids exhibited improved carcass performance while inheriting the favorable meat quality characteristics of Neijiang pigs. The results of 16S rRNA sequencing analysis showed that the relative abundance of the microbiota was similar to that of NN pigs. LDA effect size (LEfSe) results showed that *Streptococcus*, *Treponema*, *probable_genus_10* and *Fibrobacter* were the differentially enriched taxa in YN pigs (*p* < 0.05). Correlation analysis was performed on carcass, meat quality and intestinal microbiota screened out by LEfSe. The results showed that *Akkermansia* tended to positively associate with body length and oblique length in YN pigs; *Dialister* correlated positively with dressing rate and pH_45min_; *Treponema* showed positive trends with a*_45min_ and a*_24h_ (*p* < 0.05). Finally, the correlation network model preliminarily mapped associations among production traits, gut microbiota, and Kyoto Encyclopedia of Genes and Genomes (KEGG) pathways for exploratory screening. Nine core microbial taxa exhibited close correlations with phenotypic indicators, which implied that these microbes might modulate metabolic pathways to shape pig performance. Overall, hybrids inherited superior parental carcass and meat quality but harbored unique gut microbial communities relative to purebreds—these preliminary correlative observations generate new hypotheses that gut microbiota may contribute to heterosis-associated phenotypic advantages, which require further targeted validation.

## Introduction

The Neijiang pig, a local swine breed from Southwest China, is primarily distributed in the eastern Sichuan Basin at altitudes ranging from 270 to 810 m (Zhou, 2024). It is renowned for its stable genetic performance, high intramuscular fat (IMF) content, and superior meat quality, which endows it with distinct local characteristics (Long et al., 2024; Yang et al., 2024b). As the first local pig breed recognized by national standards in China, Neijiang pig’s genetic resource protection has always been highly valued by the government (Zhou, 2024). To better protect and develop the high-quality local pig breed resources in China, some studies have been carried out from fitting the growth curve of Neijiang pig, optimizing the semen freezing process, and comparing the phenotype and genome with other local pig breeds, so as to better realize the conservation and development and utilization of Neijiang pig (Chen et al., 2023a, 2023b; Liu et al., 2023; Zhang, 2022).

Cross-breeding is widely used to enhance growth, reproduction, and disease resistance in livestock (Zhu et al., 2024a). In Sichuan, Dan line and Canadian line Yorkshire pigs are widely used as parents in the production of commercial pigs (Dan et al., 2024). The Yorkshire (Large White) pig, a globally prevalent lean-meat breed, is valued for its rapid growth, high feed efficiency, and strong reproductive capacity. However, its poor disease resistance and poor meat quality are mainly manifested in low pH_45min_ value, reduced IMF, and poor marbling (MBL) scores (Gao et al., 2019).

Intestinal microorganisms can play a key role in regulating various physiological functions such as host digestion, metabolism, and immunity (He et al., 2025a). The application of 16S rRNA gene sequencing has become a cornerstone for investigating the diversity and composition of the intestinal microbiota in animals, significantly advancing research in this field (Liu et al., 2020). Several studies have pointed out that significant gut microbiota disparities exist between local and imported pigs, offering mechanistic insights into breed-specific productivity differences (Huang et al., 2024; Wu et al., 2024). Studies have shown that gut microbes can improve meat quality by regulating muscle fiber type conversion and lipid metabolism (He et al., 2025b; Liu et al., 2024b; Yang et al., 2025). Given the established influence of microbial communities on meat quality, understanding the gut microbiota of indigenous pig breeds is crucial (Kumar et al., 2025). However, the gut microbiota of the Neijiang pig—a valued Chinese genetic resource—remains poorly characterized. To address this, we compared the carcass traits, meat quality, and fecal microbial communities of 150-day-old purebred Neijiang (NN), Yorkshire (YY), and their crossbred (YN) pigs. This study aimed to elucidate how the gut microbiota influences hybrid meat quality and to identify microbial biomarkers associated with superior phenotypes, thereby providing a theoretical basis for future microbiome-driven breeding strategies.

## Materials and Methods

### Ethics statement

To ensure the highest standards of animal welfare, all experimental procedures strictly followed the guidelines of and were approved by the Animal Ethical and Welfare Committee (AEWC) of Sichuan Agricultural University (Approval No. 20220561).

### Animals and sample collection

To avoid the batch effect, the experimental pigs were from the same batch of pigs in the same pig farm of a company in southwest China, including nine NN pigs, six YN pigs and seven YY pigs. The hybrid cross was established using the Neijiang pig as the dam line and the Yorkshire pig as the sire line. All pigs were raised in the same breeding environment, adopted the same feeding mode, and fed the same batch of the same feed. Throughout the trial, pigs were fed *ad libitum* a meal-form diet formulated to meet the Neijiang pig nutrient specifications (DB5110/T 77-2024), which consisted of a corn-soybean meal base supplemented with 8% wheat bran and 3% rapeseed meal to match the breed’s higher crude-fiber tolerance, and contained no in-feed antibiotics or zinc oxide (3.0–3.2 Mcal DE kg^−1,14^–16% CP, 0.75–0.85% SID lysine, 0.70% Ca, and 0.60% P). It should be noted that individual daily feed intake and specific growth rates were not monitored in this experiment, which is acknowledged as a limitation when interpreting the microbiota and meat quality data. When the feeding age reached 150 days, the carcass and meat quality of NN, YY and YN pigs were measured (each breed consisting of three castrated males and three females, for a total of six heads per breed). All experimental procedures involving animals were approved by the Institutional Animal Welfare and Ethics Committee of Sichuan Agricultural University (Approval No. 20220561) and were conducted in strict compliance with national guidelines and the ARRIVE guidelines 2.0. Slaughter determination was carried out according to the ‘Technical Specification for Determination of Carcass Traits of Lean Meat Type Pigs’ (NY/T825-2004). Pigs were fasted for 24 h before slaughter, drank water normally, and avoided fighting. The process was performed by certified personnel in a professional slaughterhouse to minimize distress. The left longissimus dorsi muscle (LDM) were collected. Prior to slaughter, fresh rectal feces were collected from all 22 pigs (NN9, YN6, YY7). While all animals provided fecal samples, only 18 pigs (NN = 6, YN = 6, YY = 6, with a balanced sex ratio of three males and three females per breed) were processed for carcass and meat quality evaluation due to practical limitations at the slaughter facility. All samples were immediately stored in liquid nitrogen tanks and later frozen at −80 °C in a refrigerator upon return to the laboratory for subsequent Intestinal microbial analysis.

### Carcass and meat quality measurement

Post-slaughter, the carcass weight was recorded immediately. The dressing percentage was calculated as the percentage of carcass weight to slaughter body weight. Additionally, carcass length and oblique length were documented. The average backfat thickness was the average value of the three points at the thoracolumbar junction, the lumbar-spine junction and the thickest shoulder. The loin eye area was measured at the last rib using vernier calipers (height × width × 0.7 cm^2^). Subsequently, the left side of each carcass was dissected into its primary components: lean meat, fat, bone, and skin, for further analysis of tissue composition. The ham rate is the percentage of the left leg-to-hip weight to the left carcass weight cut vertically between the reciprocal first lumbar vertebra and the reciprocal second lumbar vertebra. LDM samples for meat quality traits were collected from near the last rib on the left side of the carcass within 45 min after slaughter. The LDM samples (thickness of about 5 cm) from the first to the second thoracic vertebrae were taken, and the pH, electrical conductivity, meat color at 45 min and water holding capacity (WHC, pressurized water loss) at 45 min postmortem. Subsequently, the LDM samples were stored at 4 °C, and measured again 24 h after slaughter.

### 16S rRNA sequencing and data analysis

Total DNA was extracted from pig intestinal fecal samples by CTAB/SDS method, and the purity and concentration of DNA were detected by 1% agarose gel electrophoresis. The extracted DNA samples were diluted with sterile water to a concentration of 1 ng/μL. The diluted genomic DNA was used as a template for PCR amplification. The hypervariable V3–V4 region of the 16S rRNA gene was amplified using the primer pair 314F-805R. PCR amplification was performed using Phusion^®^ High-Fidelity PCR Master Mix (New England Biolabs, Ipswich, MA, USA) following the manufacturer’s instructions. PCR products were purified by agarose gel electrophoresis on 2% agarose gels. PCR products of between 400 and 500 bp were selected for PCR product purification using GeneJET (Thermo Scientific, Waltham, MA, USA) following the manufacturer’s instructions.

QIIME2 (v2022.8.3) software and its integrated plug-in were used to analyze 16S rRNA data. First, the QIIME tool import plug-in was used to import paired-ended 16S rRNA sequencing data. Subsequently, QIIME dada2 denoising pairing plug-in and QIIME feature table filtering-feature plug-in are used to denoise and filter the original data. The filtered data uses the QIIME phylogenetic alignment to the tree’s fast tree plug-in to build a phylogenetic tree. For alpha diversity (Shannon, Simpson, and Chao1) and beta diversity (Bray–Curtis) analysis, we used the QIIME diversity core indicator plug-in. Species annotation is performed using the QIIME feature classifier plug-in. Finally, PICRUSt2 (v2.5.2) was used to predict the function of ASV abundance table (Liu et al., 2024a).

### Statistical analysis

All pork quality measurement data were organized in a matrix using Excel 2020. Statistical analyses were conducted with SPSS 26.0 and GraphPad Prism (v10.0). Regarding the statistical analysis of the slaughter performance, to reflect the overall population characteristics, the phenotypic data for each breed were pooled and analyzed collectively rather than being separated by sex. One-way analysis of variance (ANOVA) was used to analyze whether there were differences in traits among breeds. Pearson correlation analysis was used to assess the relationships between meat quality, carcass traits, and microbiota. Downstream statistical analysis and visualization of 16S data were performed in R (version 4.2.2). Differential taxa were identified using linear discriminant analysis (LDA) effect size (LEfSe) analysis with an LDA score >3.0 and a false discovery rate (FDR)-adjusted *p* < 0.05 (Benjamini–Hochberg correction based on the Kruskal–Wallis test) (Yan et al., 2025). Kyoto Encyclopedia of Genes and Genomes (KEGG) primary and secondary enrichment pathways were visualized as heat maps. KEGG three-level enrichment pathway was analyzed by STAMP software (*p* < 0.001). Pearson correlation analysis was applied to explore the potential relationships among pig phenotypes, the microbial community, and KEGG pathways. For general comparisons, statistical significance was defined as a *p* < 0.05, while a *p* < 0.01 was considered highly significant (FDR-corrected).

## Results

### Carcass characteristics

**Figure 1:**
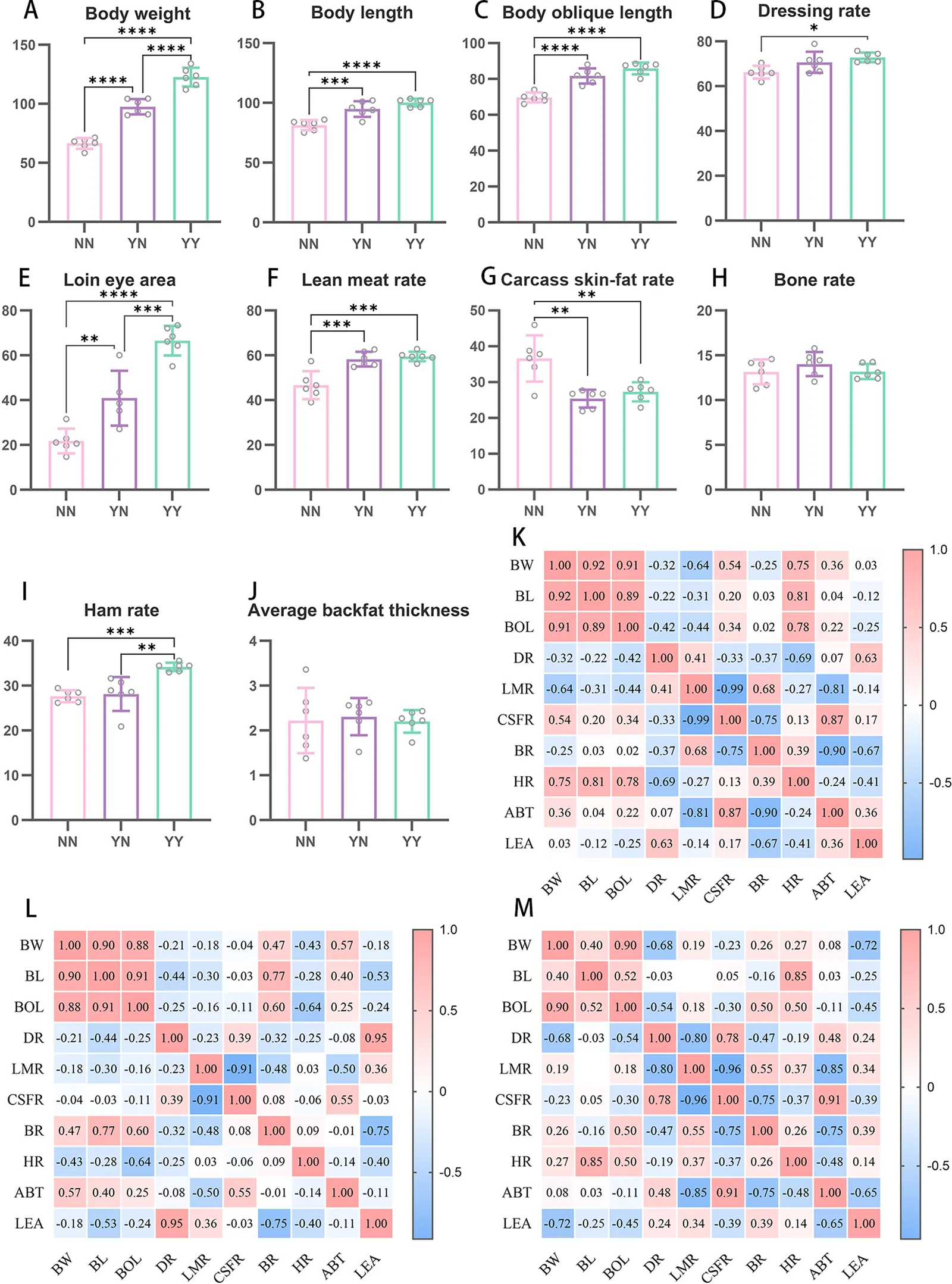
Comparative analysis of carcass traits of NN, YN and YY pigs. (A) Body weight (BW). (B) Body length (BL). (C) Body oblique length (BOL). (D) Dressing rate (DR). (E) Loin eye area (LEA). (F) Lean meat rate (LMR). (G) Carcass skin-fat rate (CSFR). (H) Bone rate (BR). (I) Ham rate (HR). (J) Average backfat thickness (ABT). (K–M) NN, YN and YY pig carcass traits correlation. Statistical significance denoted by *p < 0.05, **p < 0.01, ***p < 0.001, ****p < 0.0001 (one-way ANOVA).

The carcass traits of the three pig breeds, including body weight, body length, oblique length, dressing rate, lean meat rate, carcass skin-fat rate, bone rate, ham ratio, average backfat thickness, and loin eye area, were compared (Fig. 1). YN pigs exhibited significantly higher body weight, body length, oblique length, loin eye area, and lean meat rate compared to NN pigs, while carcass skin-fat rate was significantly lower. The body weight, loin eye area and ham ratio of YN pigs were significantly lower than those of YY pigs. There was no significant difference in bone rate and backfat thickness among the three pig breeds.

The correlation analysis of carcass traits was carried out within the three pig breeds. The correlation coefficients were positive and negative, and the correlation between the same traits or the opposite traits was higher. The correlation between carcass traits of YN pigs was similar to that of NN pigs. There was a high correlation between the three traits of body weight, body length and oblique length. There was a high positive correlation between loin eye area and dressing rate. There was also a high correlation between bone rate and body length in YY pigs, but the correlation was low in both YY and NN pigs. There was a high negative correlation between ham ratio and body weight, body length and oblique length in YN pigs, but the opposite effect was shown in NN and YY pigs. The average backfat thickness of the three pigs was positively correlated with the carcass skin-fat rate and negatively correlated with the lean meat rate.

### Meat quality traits

**Figure 2:**
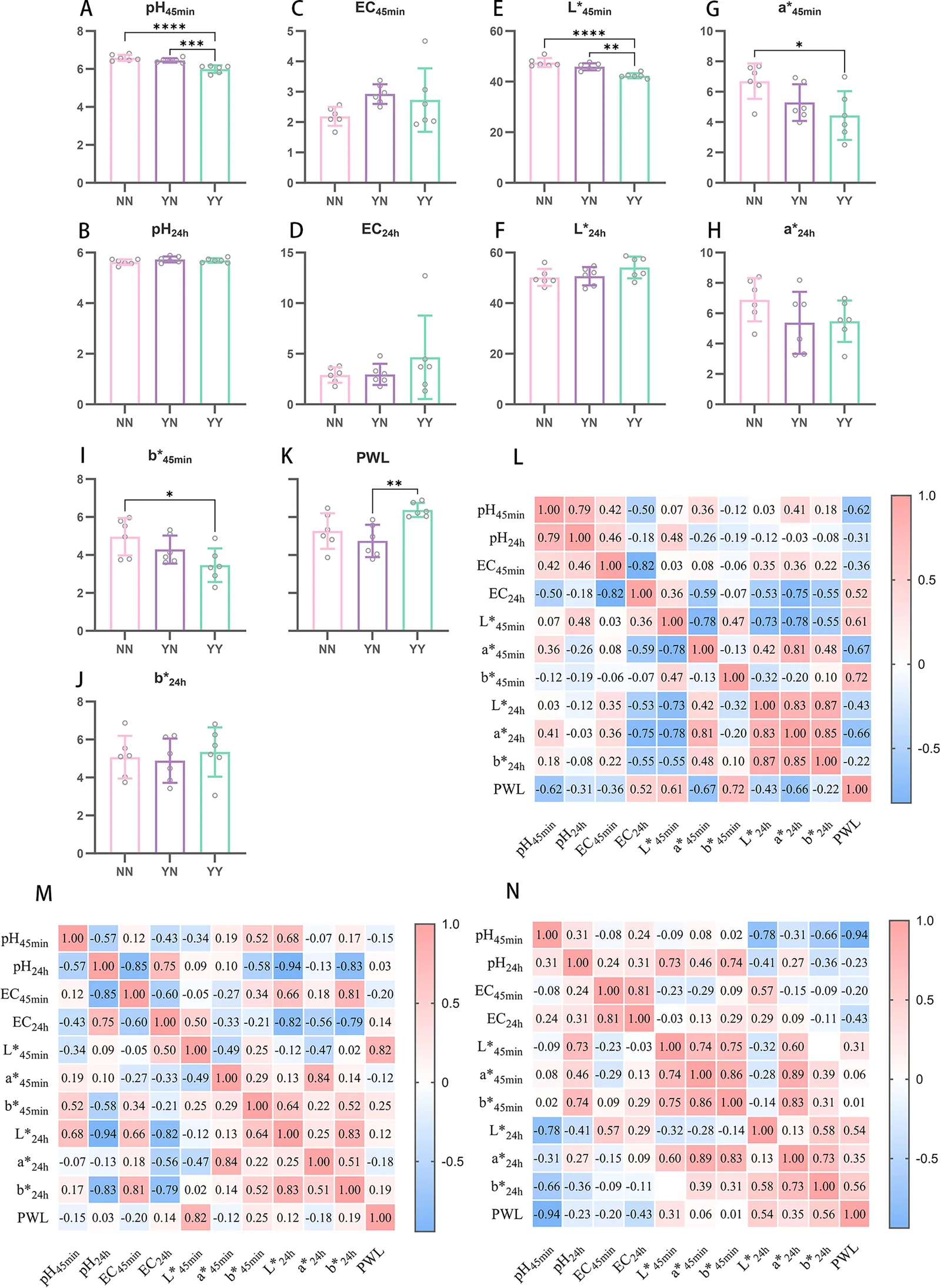
Comparative analysis of NN, YN and YY meat quality traits. (A) pH_45min_. (B) pH_24h_. (C) EC_45min_ (electrical conductivity_45min_). (D) EC_24_ (electrical conductivity_24h_). (E) L*_45min_. (F) L*_24h_. (G) a*_45min_. (H) a*_24h_. (I) b*_45min_. (J) b*_24h_. (K) PWL (pressurized water loss). (L–N) NN, YN and YY meat quality traits correlation. Statistical significance denoted by *p < 0.05, **p < 0.01, ***p < 0.001, ****p < 0.0001 (one-way ANOVA).

Figure 2 shows the meat quality traits of the three pig breeds. The pH_45min_ index of YN pigs was significantly higher than that of YY pigs, slightly lower than that of NN pigs but not significant, and there was almost no difference in pH_24h_ among the three pig breeds. The eye muscle conductivity of YN pigs measured at 45 min was higher than that of NN and YY pigs but not significant. The eye muscle conductivity data measured at 24 h showed that NN and YN pigs were lower than YY pigs. In the meat color index, the L*_45min_ data of YN pig was significantly higher than that of YY pig, and there was no significant difference between YN pig and the other two pig breeds in other meat color indexes. The pressure water loss rate of YN pigs was lower than that of the other two pigs, and significantly lower than that of YY pigs.

Similarly, we also analyzed the correlation of meat quality traits, and observed that there were some differences in the correlation patterns of meat quality traits among the three pig breeds. In a*_45min_ and a*_24h_, L*_24h_ and b*_24h_, there was a very strong positive correlation between the three pig breeds. The pH_45min_ and pH_24h_ of YN pigs showed a negative correlation, and a positive correlation with b*_45min_ and L*_24h_. PH_24h_ is positively correlated with electrical conductivity_24h_, and negatively correlated with L*_24h_, b*_24h_ and electrical conductivity_45min_. The positive correlation between electrical conductivity_45min_ and L*_24h_, b*_24h_ in YN pigs was enhanced. The correlation between electrical conductivity_24h_ and other traits was similar to that of NN pigs. electrical conductivity_24h_ showed a high negative correlation with electrical conductivity_45min_, L*_24h_, a*_24h_ and b*_24h_. However, YN pigs showed a negative correlation between electrical conductivity_24h_ and pH_24h_, which was the same as YY pigs. The correlation pattern between L*_45min_ and a*_45min_, b*_45min_, L*_24h_, a*_24h_, b*_24h_ in YN pigs was consistent with that in NN pigs, but the degree of correlation was weakened. There was a positive correlation between L*_45min_ and electrical conductivity_24h_, pressurized water loss in YN pigs, and the correlation was enhanced compared with NN and YY pigs. The correlation between a*_45min_ and pressurized water loss in YN and YY pigs was weak. Between a*_45min_ and electrical conductivity_24h_ and between a*_45min_ and L*_45min_, NN pigs and YY pigs showed opposite correlation characteristics, and YN pigs showed negative correlation with the same traits as NN pigs, but the degree of correlation was reduced. NN pigs showed a high correlation between b*_45min_ and pressurized water loss and between L*_24h_ and a*_24h_. There was a positive correlation between b*_45min_ and L*_24h_ in YN pigs. YY pigs showed a high positive correlation between b*_45min_ and a*_24h_. NN pigs and YN pigs showed a high positive correlation between L*_24h_ and b*_24h_.

### 16S rRNA sequencing data and alpha, beta diversity analysis

**Figure 3:**
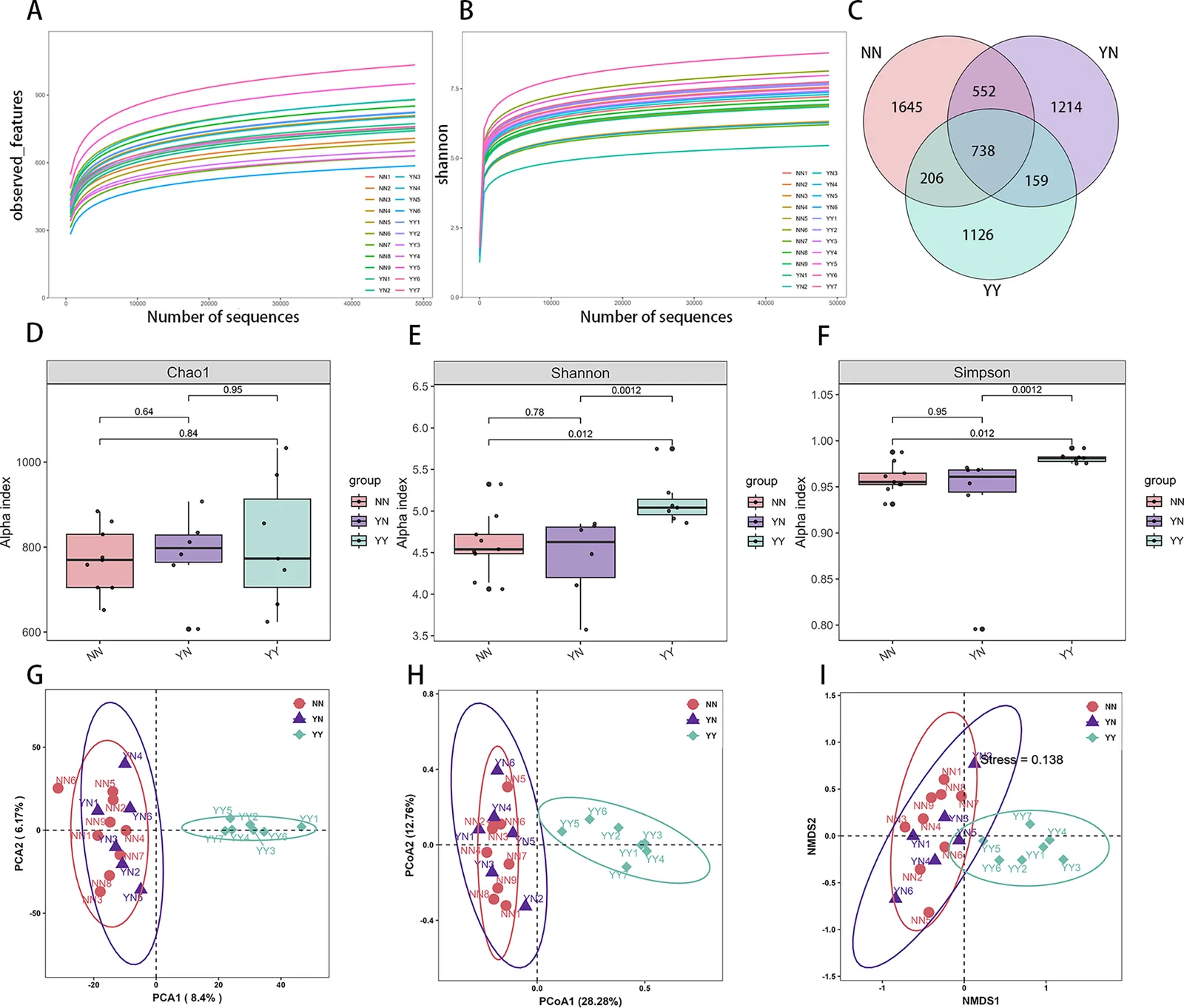
Quality and diversity analysis results of 16S rRNA sequencing data. (A) Observed features. (B) Shannon diversity index. (C) Venn diagram of feature count distribution in different pig breeds. (D–F) Alpha diversity indices: Chao1 index, Shannon index and Simpson index. (G–I) Beta diversity indices: PCA plots, PCoA map based on Bray–Curtis distance, NMDS plots (ellipses represent 95% confidence intervals).

In order to reveal the differences of intestinal microbes in different breeds of pigs, fresh fecal samples were collected from three breeds of pigs at 150 days of age, and a total of 22 samples (NN9, YN6, YY7) were collected. After quality control and clustering, a total of 5,762 feature counts were obtained. In order to reflect the species richness and uniformity of the sequencing data and the rationality of the sequencing process, the Shannon index curve was calculated. The curve results showed that the samples in each group entered the plateau phase, indicating that the current sequencing depth could detect most of the species in the sample and could meet the subsequent analysis requirements (Figs. 3A and 3B). The Venn diagram shows the number of feature count shared and unique among the three pig breeds (Fig. 3C). There were 738 feature counts in the three pigs, 1,645 feature counts were unique to NN pigs, 1,214 feature counts were unique to YN pigs, and 1,126 feature counts were unique to YY pigs. The alpha diversity analysis of the microbial community of the three breeds of pigs was carried out, and the microbial species in a single sample and the corresponding proportion of species were observed. Shannon, Simpson, and Chao1 indices were calculated to evaluate richness and evenness (Figs. 3D–3F). The results showed that the Shannon and Simpson indexes of NN and YN pigs were similar, which were significantly lower than those of YY pigs. There was no significant difference in Chao1 index among the three pig breeds. β diversity can measure the similarity of microbiota composition between different samples. In order to evaluate the degree of difference in bacterial community structure among the three breeds of pigs, principal component analysis (PCA), principal coordinate analysis (PCoA) and non-metric multidimensional scaling (NMDS) were performed to describe the β diversity among pig breeds (Fig. 3H). As shown in the PCoA plot, the first principal component (PC1) accounted for 28.28%, and the second principal component (PC2) accounted for 12.76%. YN pigs and NN pigs were relatively close in the two principal components, while YY pigs were nearly completely separated from YN pigs in the first principal component, indicating that the intestinal function of YN pigs was close to that of NN pigs, but there was a significant difference in β diversity between YN pigs and YY pigs.

### Intestinal microbiota composition of different pigs

**Figure 4:**
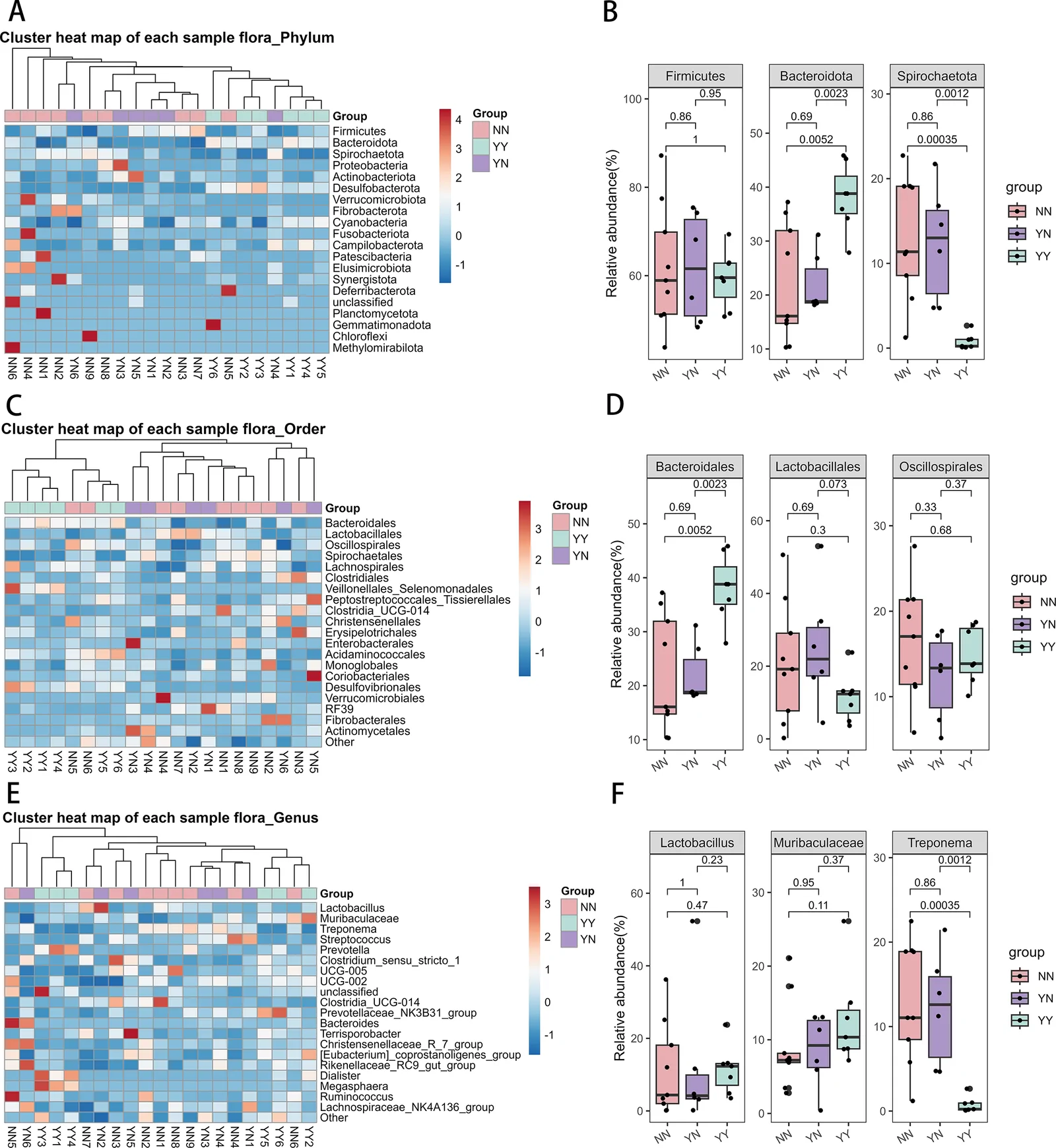
The abundance of microbial microbiota in different pig populations at the phylum and genus levels. (A) Heat map of species distribution at phylum level. (B) The first three species box diagram at the phylum level. (C) Heat map of species distribution at order level. (D) The first three species box diagram at the order level. (E) Heat map of species distribution at genus level. (F) The first three species box diagram at the genus level.

In order to determine the composition and differences of intestinal microorganisms at the Phylum and Genus levels in the three pig breeds, the abundance of intestinal microorganisms was compared. At the Phylum level (Figs. 4A and 4B), a total of 19 Phylum were identified, and the top three Phylum with relative abundance (more than 96%) were Firmicutes, Bacteroidota and Spirochaetota. Firmicutes (relative abundance 59–61%), Bacteroidota (relative abundance 22–38%) and Spirochaetota (relative abundance 0.76–13%) were dominant. In terms of the relative abundance of the microbiota, the relative abundance of Firmicutes in the three pig breeds was relatively close. The relative abundance of Bacteroidota and Spirochaetota in YN pigs and NN pigs was close, and there was a significant difference between YN pigs and YY pigs (*p* < 0.01).

At the Order level (Figs. 4C and 4D), The relative abundance of *Bacteroidales*, *Lactobacillales* and *Oscillospirales* ranked the top three. Similar to Phylum, the microbiota composition of YN pigs was closer to that of NN pigs. The relative abundance of *Bacteroidales* in YY pigs (38.1%) was significantly higher than that in NN (22.1%) and YN pigs (22.0%) (*p* < 0.01). The relative abundance of *Lactobacillales* in YN pigs (25.1%) and NN pigs (21.0%) was higher than that in YY pigs (11.5%), but not significant. The abundance of *Oscillospirales* in YN pigs (12.3%) was slightly lower than that in NN pigs (6.5%) and YY pigs (14.9%).

At the Genus level (Figs. 4E and 4F), the microbiota composition of YN pig was close to NN pig, and there was a big difference between YN pig and YY pig. In terms of relative abundance of microbiota, the dominant Genus in YN pigs were *Lactobacillus* (12.6%), *Streptococcus* (12.4%), *Treponema* (12.1%), *Muribaculaceae* (8.4%); the dominant Genus in NN pigs were *Treponema* (13.0%), *Lactobacillus* (11.3%), *Streptococcus* (9.7%) and *Muribaculaceae* (9.1%). *Prevotella* (15.2%), *Muribaculaceae* (12.8%), *Lactobacillus* (11.4%), *Dialister* (5.1%) and *Megasphaera* (4.6%) were the dominant Genus of YY pigs. There was no significant difference in the relative abundance of all genera annotated by YN pigs compared with NN pigs. In *Treponema*, *Streptococcus*, *Clostridia_UCG-014* and *Lachnospiraceae_NK4A136_group*, the relative abundance of YN was significantly higher than that of YY pigs (*p* < 0.05). In *Prevotella*, *Dialister* and *Megasphaera*, the relative abundance of YN pigs was significantly lower than that of YY pigs (*p* < 0.01). It can be seen that no matter at the Phylum level or the Genus level, the intestinal microbial composition of NN pigs and YN pigs is similar, and the intestinal microbial composition of YY pigs is unique.

**Figure 5:**
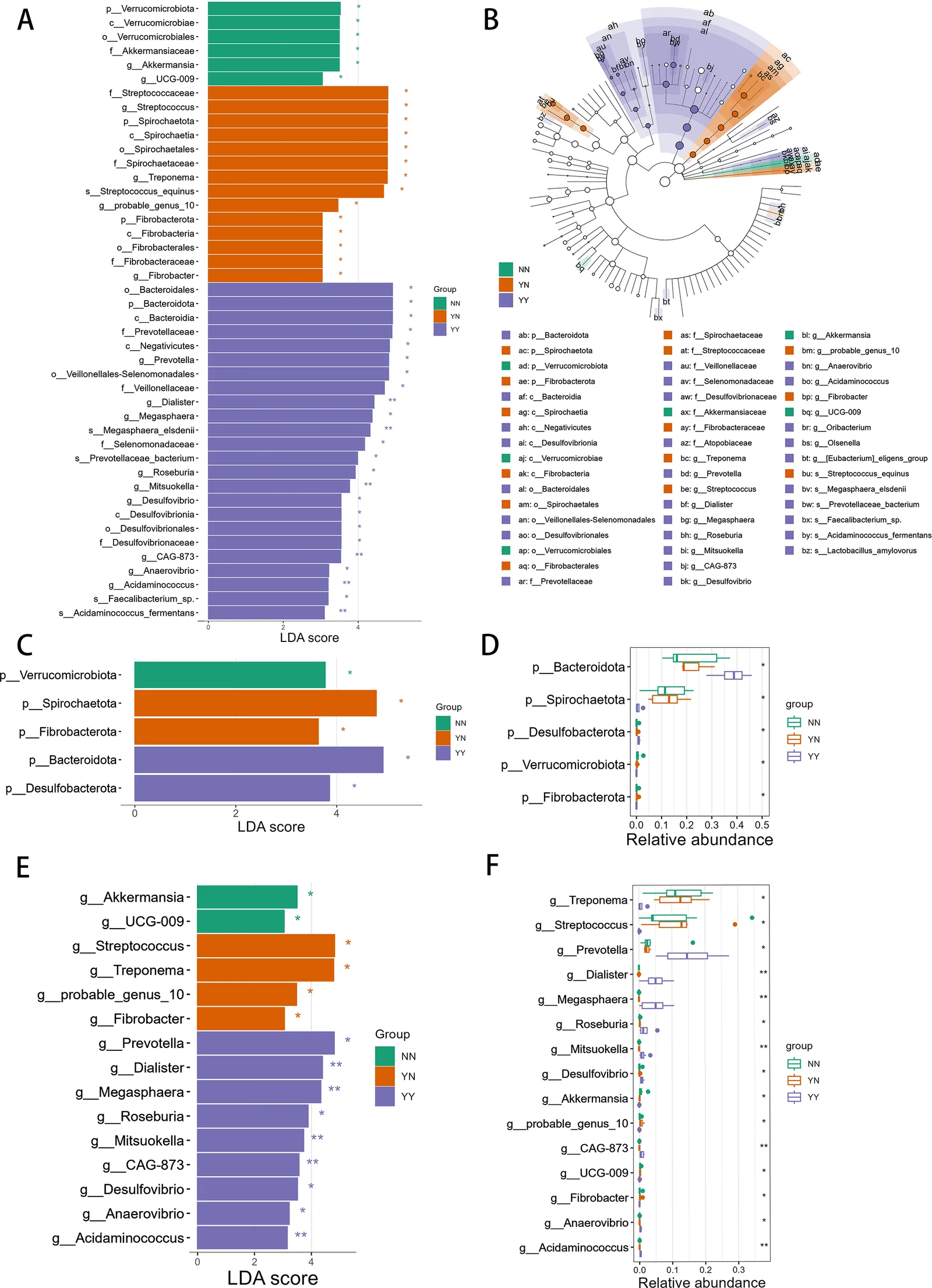
LEfSe difference analysis of NN, YN and YY pigs. (A) LDA score >3 distribution bar chart. (B) Taxonomic cladogram obtained from linear discriminant analysis effect size (LEfSe) sequence analysis. Species taxonomy from phyla to species (inside–outside). The diameter of each circle represents the relative abundance of the taxon, and the color corresponds to the grouping. (C) Phylum level LDA score >3 distribution bar chart. (D) The relative abundance of bacteria with Phylum level LDA score >3. (E) Genus level LDA score >3 distribution bar chart. (F) The relative abundance of bacteria with Genus level LDA score >3 (*p < 0.05, **p < 0.01).

To identify specific microbial taxa with significant differences in abundance among the intestinal microbiota of different pig breeds, LEfSe was performed. This method combines statistical significance with biological relevance to evaluate the effect size of differentially abundant features. Taxa with an LDA score >3 (Fig. 5A) were considered significantly different. A total of 44 potential biomarkers were identified across the three pig breeds (6 in NN pigs, 14 in YY pigs, and 24 in YN pigs). At the genus level, *Streptococcus*, *Treponema*, *probable_genus_10* and *Fibrobacter* were significantly enriched in YN pigs. *Akkermansia* and *UCG-009* were identified as significantly enriched taxa in NN pigs. Additionally, *Prevotella*, *Dialister*, *Megasphaera*, *Roseburia*, *Mitsuokella*, *CAG-873*, *Desulfovibrio*, *Anaerovibrio* and *Acidaminococcus* were significantly enriched in YY pigs (Fig. 5E).

### Prediction of intestinal microbial function in different pigs

**Figure 6:**
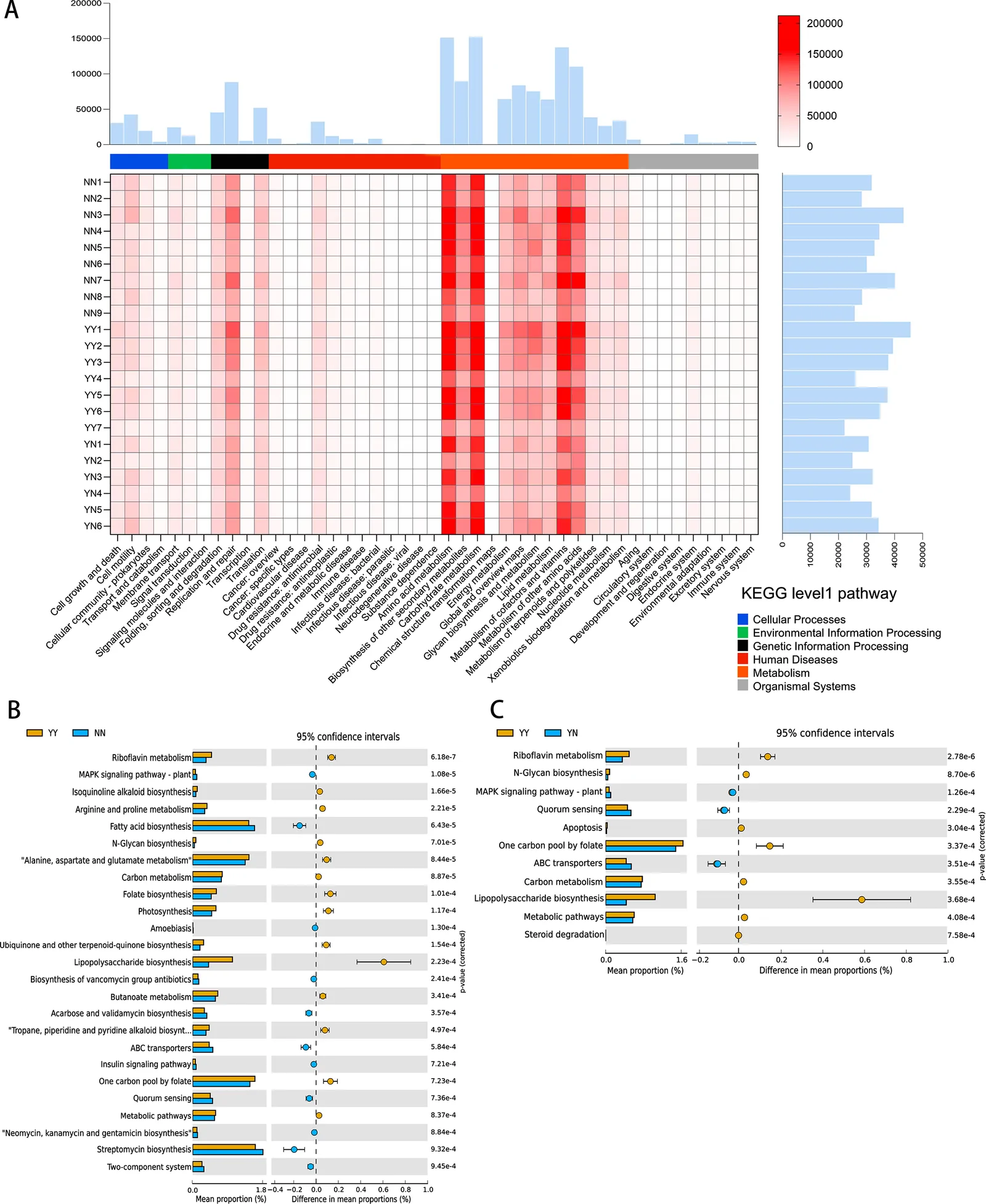
Prediction results of intestinal microbial function based on KEGG. (A) KEGG database level 1 and level 2 functional annotation heat map. (B, C) Functional prediction differences for NN *vs*. YY and YN *vs*. YY in KEGG database level 3 pathways (*p* < 0.001).

Further mining of 16S sequencing data, PICRUSt2 was used to predict the possible function of bacterial genes. The intestinal microbial function of pigs was predicted based on the KEGG database (Fig. 6A). The results of the secondary pathway showed that most of the predicted pathways in the three pig breeds were associated with Metabolism (18 pathways) and Genetic Information Processing (three pathways). The KEGG level 3 pathway was further analyzed. When *p* < 0.001, 25 pathways were found between NN and YY, and 19 pathways (76%) were related to metabolism (Fig. 6B). Eleven pathways were found between YN and YY, and seven pathways (64%) were related to metabolism (Fig. 6C). The main metabolic pathways are clustered in amino acid metabolism, carbohydrate metabolism, metabolism of cofactors and vitamins, biosynthesis of other secondary metabolites. Lipopolysaccharide biosynthesis, one carbon pool by folate and riboflavin metabolism were observed to be abundant in YY pigs. Compared with YY pigs, ABC transporters, quorum sensing and mitogen-activated protein kinase (MAPK) signaling pathway-plant were abundant in NN and YN pigs. In addition, streptomycin biosynthesis, fatty acid biosynthesis, acarbose and validamycin biosynthesis were also abundant in NN pigs.

### Correlation analysis between phenotypic data and intestinal microbiota

**Figure 7:**
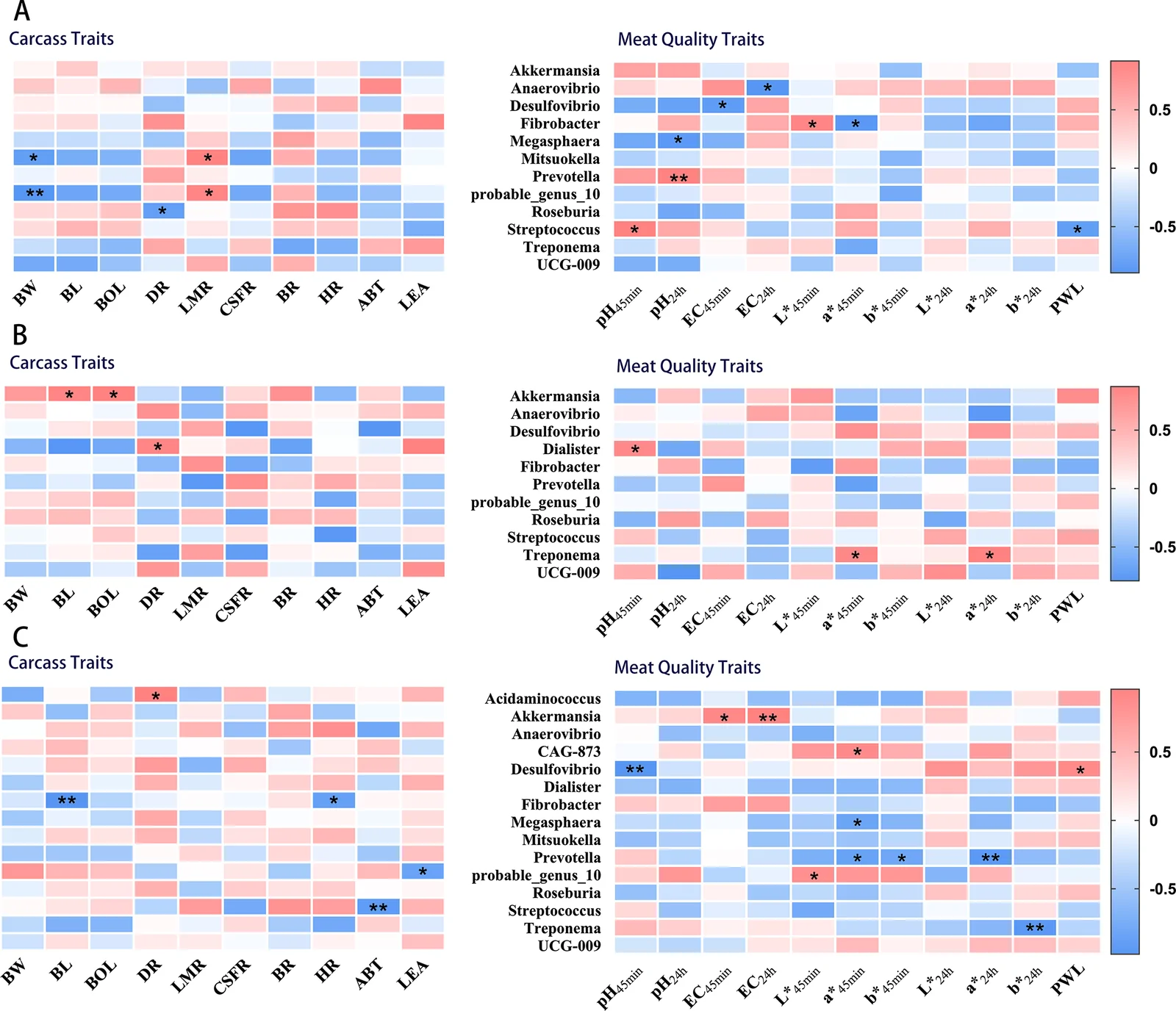
Correlation analysis between intestinal microbiota and different phenotypic characteristics. (A) NN pig. (B) YN pig. (C) YY pig (*p < 0.05, **p < 0.01).

To investigate the potential associations between intestinal microbial composition and carcass or meat quality traits across different pig breeds, Pearson correlation analysis was performed. Overall, the microbial correlation profiles of hybrid YN pigs differed distinctly from those of purebred NN and YY pigs. In YN pigs (Fig. 7B), *Akkermansia* was highly correlated with growth traits (body weight, body length, oblique length, bone rate) and several meat quality indices (L*_45min_, pressurized water loss). Additionally, dressing rate and pH_45min_ were positively correlated with *Dialister*. *Treponema* showed a significant positive correlation with color stability (a*_45min_, a*_24h_) but negatively correlated with dressing rate, carcass skin-fat rate, average backfat thickness, and electrical conductivity_24h_. In NN pigs (Fig. 7A), body weight was significantly negatively correlated with *Mitsuokella* and *probable_genus_10*, whereas loin meat rate showed a positive correlation with these two genera. Dressing rate was negatively correlated with *Roseburia*. Regarding meat quality, five specific microbial-metric pairs exhibited significant negative correlations: pH_24h_ with *Megasphaera*, electrical conductivity_45min_ with *Desulfovibrio*, electrical conductivity_24h_ with *Desulfovibrio*, a*_45min_ with *Fibrobacter*, and pressurized water loss with *Streptococcus*. In YY pigs (Fig. 7C), *Fibrobacter* was negatively correlated with body length and ham rate, while *Streptococcus* was inversely correlated with average backfat thickness. Dressing rate was positively correlated with *Acidaminococcus*. Notably, *probable_genus_10* was negatively correlated with loin eye area but positively with L*_45min_. Furthermore, *Akkermansia* and *CAG-837* were positively correlated with electrical conductivity and a*_45min_, respectively. *Desulfovibrio* was positively correlated with pressurized water loss but negatively with pH_45min_. Finally, both *Megasphaera* and *Prevotella* showed negative correlations with a*_45min_; *Prevotella* also showed negative correlations with b*_45min_ and a*_24h_, while *Treponema* was negatively correlated with b*_24h_.

### Correlation analysis of phenotype-gut microbiota-KEGG pathway

**Figure 8:**
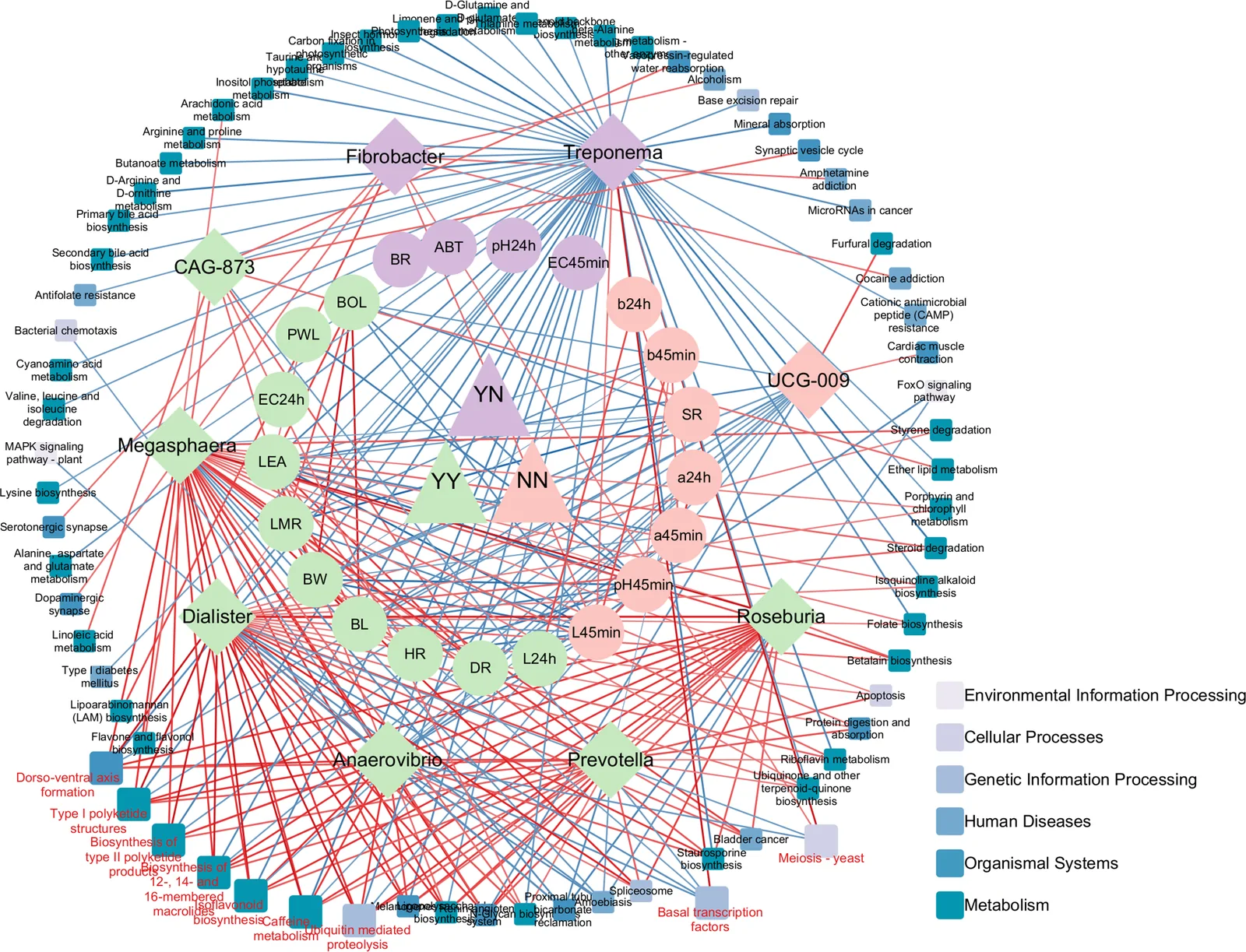
Correlative network model of host phenotypes (carcass and meat quality traits) with intestinal microbiota, and between microbiota and KEGG pathways. The triangle represents the pig breed, the circle represents the phenotype, the square represents the flora, and the round rectangle represents the KEGG pathway. The color of the trait and the flora indicates that it is highly expressed in the corresponding pig breeds. The strength of the interaction is represented by the thickness and depth of the lines. The stronger the interaction, the thicker and darker a line is. The red line represents the positive relationship, and the blue line represents the negative relationship (|*r*| > 0.5, *p* < 0.05).

To explore the potential complex relationship between pig phenotype, microbial community and KEGG pathway, a Pearson-based correlation network was constructed to visualize their multi-level relationships (Fig. 8). Results indicated that nine LEfSe-screened genera were strongly correlated with both phenotypic traits and 71 KEGG pathways. Among these, nine bacteria-KEGG pairs exhibited the most robust correlations (|*r*| > 0.9, *p* < 0.05). These included *Treponema* associated with Basal transcription factors and Meiosis-yeast, and *Dialister* correlated with Ubiquitin-mediated proteolysis, Dorso-ventral axis formation, and several metabolic pathways (caffeine metabolism; isoflavonoid biosynthesis; biosynthesis of 12-, 14-, and 16-membered macrolides; biosynthesis of type II polyketide products; Type I polyketide structures).

## Discussion

The gut microbiota significantly influences host physiology, yet its specific impact on porcine meat quality and regulatory mechanisms remains unclear (Liang et al., 2024; Yang et al., 2025). The Neijiang pig, a valuable local breed in Sichuan, China, possesses excellent meat characteristics but suffers from limited commercial viability due to low lean meat and feed efficiency (Yang et al., 2003). To explore the microbiological basis of these traits, we compared the carcass performance, meat quality, and fecal microbiota of purebred Neijiang, Yorkshire, and crossbred pigs. Uncovering these microbiota-phenotype interactions may unlock new approaches for breed improvement and conservation.

NN pigs exhibited significantly lower growth and carcass metrics—including body weight, body length, oblique length, dressing rate, loin eye area, lean meat rate, and ham ratio—compared to YY pigs, while demonstrating a significantly higher carcass skin-fat rate than both YY and YN pigs. YN pigs were significantly better than NN pigs in body weight, body length, oblique length, loin eye area and lean meat rate, indicating that crossbreeding effectively enhances the meat production capacity of Neijiang pigs. This aligns with the typical heterosis observed in hybrid breeding programs. Specifically, the loin eye area—a key indicator for rapidly assessing carcass merit—was improved in YN pigs compared to NN pigs. Consistent with previous findings (Guo, 2024), loin eye area showed a significant positive correlation with lean meat rate in both YY and YN pigs. Overall, these results demonstrate that crossbreeding successfully ameliorates the carcass deficiencies of purebred NN pigs.

Meat color and pH are critical sensory and physicochemical attributes used to assess pork freshness and quality (Chodová et al., 2021; Zhu et al., 2022). Compared to commercial Western breeds like Yorkshire, Chinese indigenous pigs typically exhibit slower growth and higher fat deposition, but yield superior meat quality (Tao et al., 2017). In our study, meat color profiles differed significantly among the breeds. At 45 min post-slaughter, NN pigs exhibited the highest a* value (redness), followed by YN pigs, whereas YY pigs presented the palest color. This aligns with consumer preferences, as pork with a higher L* value (lightness) and lower a* value is generally perceived as less desirable (Yang et al., 2024a). Furthermore, from 45 min to 24 h post-slaughter, L*, a*, and b* values increased across all groups; however, YY pigs showed the most pronounced discoloration, while YN pigs maintained remarkable color stability with only a slight, non-significant increase. Regarding pH, a rapid drop post-slaughter is undesirable. In our data, compared with YY pigs, the pH_45min_ data of YN pigs was significantly improved. There are differences in meat color among different pig breeds. Beyond color and pH, WHC is another essential metric, as moisture retention directly influences juiciness, shear force, and shelf-life during transportation and storage (Matarneh, Silva & Gerrard, 2021). Using the pressurized water method (Li et al., 2023), we found that YN pigs had significantly lower pressurized water loss compared to YY pigs. This indicates improved WHC in the hybrid YN pigs, which contributes to their better meat quality. Therefore, the hybridization strategy not only improves growth performance but also enhances key meat quality attributes, positioning YN pork as a higher-quality product.

A growing body of evidence highlights the critical role of gut microbiota in porcine health and physiology (Patil, Gooneratne & Ju, 2020). Notably, microbial composition varies significantly between pig breeds, particularly when comparing lean-type overseas breeds with Chinese indigenous breeds characterized by higher adiposity (Alain et al., 2014). In alignment with this, our analysis revealed a distinct structural divergence: the hybrid YN pigs clustered closely with the native NN pigs, both being clearly separated from the overseas YY pigs. Overall, Firmicutes, Bacteroidota, and Spirochaetota were the dominant phyla, with *Lactobacillus*, *Muribaculaceae*, and *Treponema* being the most abundant genera. The dynamic balance between Firmicutes and Bacteroidota is crucial for maintaining host gut microecology (Wang et al., 2024). As the predominant phylum in the mammalian gut, Firmicutes is primarily associated with fat metabolism and energy absorption (Gao et al., 2019). In YY pigs, in addition to Firmicutes, the abundance of Bacteroidota was also very high and significantly higher than that of NN and YN pigs. The decreased abundance of Firmicutes and the increased abundance of Bacteroidota in pigs can be used as an indicator of intestinal inflammation or cancer (Zhao et al., 2023). Interestingly, YY pigs exhibited a distinct phylum-level shift, displaying a significantly higher abundance of Bacteroidota compared to both NN and YN pigs. Given that a decreased Firmicutes/Bacteroidota ratio has been recognized as a microbial signature associated with gut inflammatory response (Zhao et al., 2023), this specific microbial shift in YY pigs may reflect underlying differences in intestinal metabolic profiles and inflammatory status compared to the indigenous breeds.

Beyond phylum-level differences, distinct genus-level shifts further delineated the breed-specific microbiota profiles. Lean-type YY pigs harbored a significantly higher abundance of *Prevotella* (15.1%) compared to NN (3.9%) and YN (2.4%) pigs. Despite its well-established role in fiber degradation, the enrichment of *Prevotella* in YY pigs aligns with hypotheses linking its proliferation to enhanced energy absorption and rapid growth (Amat et al., 2020; Chen et al., 2021). This growth-promoting phenotype in YY pigs was further supported by a marked enrichment of short-chain fatty acids (SCFA)-producing genera, including *Megasphaera* and *Dialister* (Ma et al., 2024). The substantial SCFA supply generated by these taxa provides a plausible microbial mechanism for the superior growth performance and feed efficiency observed in YY pigs, which may, however, come at the expense of meat quality. Conversely, the indigenous NN and hybrid YN pigs exhibited a distinct microbial signature characterized by an enrichment of *Spirochaetota* and *Treponema*. Consistent with previous findings in obese or higher-adiposity models (Yan et al., 2016), the elevated *Spirochaetota* in NN and YN pigs likely reflects their genetic predisposition for fat deposition. Furthermore, the higher abundance of *Treponema* in these breeds echoes observations in Min pigs, potentially conferring a superior capacity for crude fiber digestion (Zhang et al., 2018). Notably, NN and YN pigs also demonstrated a significant enrichment of several health-modulating and barrier-protective taxa. For instance, *Streptococcus*, often recognized as an opportunistic pathogen (Ferrando & Schultsz, 2016), exhibited a significantly higher relative abundance in NN and YN pigs. Rather than indicating a pathological state, this enrichment may reflect a context-dependent metabolic role, as specific *Streptococcus* strains are known to modulate bile acid composition and subsequently reduce fat deposition (Luo et al., 2022). This protective metabolic profile was further augmented in YN pigs by the significant enrichment of *Lachnospiraceae_NK4A136_group* and *Clostridia_UCG-014*. Both genera are recognized as beneficial commensals that promote SCFA synthesis, repair colonic barrier damage, and suppress inflammatory responses (Gao et al., 2023; Zhu et al., 2024b). The positive association of *Clostridia_UCG-014* with body weight (Rhayat et al., 2023) suggests that YN pigs may uniquely leverage these beneficial taxa to enhance nutritional absorption and gut health, thereby mitigating the growth limitations typically seen in pure indigenous breeds.

To elucidate the functional potential of the breed-specific microbiota, we performed functional prediction using PICRUSt2. KEGG level 3 analysis revealed distinct metabolic profiles among the breeds, with NN and YN pigs clustering together and diverging significantly from YY pigs. Notably, pathways related to ABC transporters, quorum sensing, and the MAPK signaling pathway (plant) were significantly enriched in NN and YN pigs. Given that ABC transporters are closely associated with lipid transport, pathogen immune responses, and oxidative stress (Guan et al., 2023), this shared functional enrichment may partially explain the unique meat quality characteristics and stress resistance observed in the indigenous and hybrid breeds.

Finally, correlation network analysis preliminarily indicated that the overall associations among carcass traits, meat quality, and intestinal microbiota in YN pigs differed markedly from those in NN and YY pigs. Notably, all microbiota–phenotype and microbiota–KEGG correlation analyses in this section are exploratory without FDR correction for multiple testing, and formal statistical verification with correction is needed in follow-up research. Specifically, the longer body length and oblique length observed in YN pigs were positively associated with the abundance of *Akkermansia*. Furthermore, redness values (a*_45min_ and a*_24h_) were positively correlated with *Treponema*, while pH_45min_ displayed a strong association with *Dialister*. The correlation network further linked these phenotype-related taxa to predicted metabolic pathways inferred from 16S rRNA data *via* PICRUSt2, rather than direct *in vivo* measurement of gene expression. *Dialister* correlated with a set of pathways, including caffeine metabolism, 12-, 14-, isoflavonoid biosynthesis, biosynthesis of type II polyketide products and type I polyketide structures. Additionally, *Dialister* showed strong positive relationships with dorso-ventral axis formation and ubiquitin-mediated proteolysis pathways. Given that *Dialister* participates in fiber degradation and SCFA production (Rahman et al., 2023), these diverse pathway linkages may reflect its multifaceted involvement in host metabolism. Similarly, *Treponema* exhibited strong positive correlations with basal transcription factors and the meiosis-yeast pathway. It should be noted that predicted pathways such as dorso-ventral axis formation, meiosis-yeast and plant MAPK signaling are not canonical pathways regulating pig carcass and meat quality, and their biological functions in porcine phenotypes require dedicated functional assays for confirmation. Collectively, these correlation results originate from a limited sample cohort. These associations suggest potential microbiota–phenotype relationships that require validation in larger cohorts and mechanistic studies.

## Conclusion

In conclusion, although the hybrid YN pigs retained a gut microbial profile closely resembling that of the purebred Neijiang pigs, they exhibited significant improvements in key production metrics, such as body weight, body length, and loin eye area. Correlation analysis highlighted specific microbiota-phenotype axes driving these improvements; for instance, the enrichment of *Dialister*, *Treponema*, and *Akkermansia* in YN pigs was selectively associated with carcass dimensions, pH, and meat color, respectively. To our knowledge, this is the first study to systematically compare the carcass performance, meat quality, and underlying microbial regulatory mechanisms among Neijiang, Yorkshire, and their hybrid offspring, providing a valuable reference for the local pig breeding industry in western China. However, several limitations of this study should be acknowledged. First, the relatively small sample size (*n* = 6 per breed) limits statistical power, necessitating cautious interpretation to avoid false positives driven by individual outliers. Second, due to the multiple pairwise correlations performed without false discovery rate (FDR) correction, the reported p should be viewed strictly as preliminary and exploratory. These correlations are intended to highlight potential trends for future hypothesis generation rather than to infer definitive mechanistic relationships. Future studies utilizing larger cohorts are warranted to validate these findings, enable robust multivariate analyses (*e.g*., incorporating sex as an interacting variable), and establish causal links between specific gut microbes and meat quality. Furthermore, the absence of individual daily feed intake and precise growth rate data represents a confounding factor that should be integrated into future experimental designs to better delineate the microbiota-meat quality nexus.

## Supplemental Information

10.7717/peerj.21663/supp-1 Supplemental Information 1 Raw data for carcass characteristics analysis.

10.7717/peerj.21663/supp-2 Supplemental Information 2 The 16S rRNA data analysis code involved in this study.

10.7717/peerj.21663/supp-3 Supplemental Information 3 Raw data for meat quality traits.

10.7717/peerj.21663/supp-4 Supplemental Information 4 ARRIVE checklist.

## Additional Information and Declarations

### Data Availability

The following information was supplied regarding data availability:

The data and code are available in the Supplemental Files.

The raw sequence data are available at Genome Sequence Archive: CRA028885.
